# Deficient GATA6–CXCR7 signaling leads to bicuspid aortic valve

**DOI:** 10.1242/dmm.050934

**Published:** 2024-09-10

**Authors:** Rebeca Piñeiro-Sabarís, Donal MacGrogan, José Luis de la Pompa

**Affiliations:** ^1^ Intercellular Signaling in Cardiovascular Development and Disease Laboratory, Centro Nacional de Investigaciones Cardiovasculares (CNIC), Melchor Fernández Almagro 3, 28029 Madrid, Spain; ^2^ Ciber de Enfermedades Cardiovasculares, Instituto de Salud Carlos III, Melchor Fernández Almagro 3, 28029 Madrid, Spain

**Keywords:** Bicuspid aortic valve, Outflow tract, Endocardial cushion development, Cardiac neural crest, GATA6, ACKR3/CXCR7

## Abstract

The cardiac outflow tract (OFT) transiently links the ventricles to the aortic sac and forms the arterial valves. Abnormalities in these valves, such as bicuspid aortic valve (BAV), are common congenital anomalies. *GATA6-*inactivating variants cause cardiac OFT defects and BAV, but their mechanisms are unclear. We generated *Gata6^STOP/+^* mice using CRISPR-Cas9, which show highly penetrant BAV (70%) and membranous ventricular septal defects (43%). These mice exhibited decreased proliferation and increased ISL1-positive progenitor cells in the OFT, indicating abnormal cardiovascular differentiation. *Gata6* deletion with the *Mef2c^Cre^* driver line recapitulated *Gata6^STOP/+^* phenotypes, indicating a cell-autonomous role for *Gata6* in the second heart field. *Gata6^STOP/+^* mice showed reduced OFT length and caliber, associated with deficient cardiac neural crest cell contribution, which may cause valvulo-septal defects. RNA-sequencing analysis showed depletion in pathways related to cell proliferation and migration, highlighting *Cxcr7* (also known as *Ackr3*) as a candidate gene. Reduced mesenchymal cell migration and invasion were observed in *Gata6^STOP/+^* OFT tissue. CXCR7 agonists reduced mesenchymal cell migration and increased invasion in wild-type but not in *Gata6^STOP/+^* explants, indicating the GATA6-dependent role of CXCR7 in OFT development and its potential link to BAV.

## Teaser

**Summary:** *Gata6* haploinsufficiency causes bicuspid aortic valve and congenital heart defects associated with reduced second heart field progenitor differentiation, proliferation and migration, impaired endocardial cushion development and deficient CXCR7 signaling.

## INTRODUCTION

Congenital heart defects are the commonest birth defects (Hoffman and Kaplan, 2002; van der Linde et al., 2011). Up to 30% of all congenital heart defects involve the outflow tract (OFT) (Srivastava and Olson, 2000; Thom et al., 2006), which briefly connects the embryonic ventricles to the aortic sac at the arterial pole of the developing heart (Rochais et al., 2009). OFT formation relies on the timely contribution of progenitor cell populations from within and outside the cardiac developmental field. Defective OFT development leads to the misalignment of the aorta and pulmonary trunks, and failure to separate systemic and pulmonary circulations (Conway et al., 2003). The endocardial cushions in the OFT are formed via the epithelial–mesenchymal transition of the endocardium and the invasion of extra-cardiac neural crest cells (cNCCs) (Snarr et al., 2008). The latter migrate into the caudal pharyngeal arches to septate the OFT and contribute to valve leaflet patterning, giving rise to the arterial (aortic and pulmonary) valves (Lin et al., 2012; Phillips et al., 2013; Snarr et al., 2008).

Bicuspid aortic valve (BAV) is the commonest congenital heart defect, affecting up to 0.5 to 2% of the population (Masri et al., 2017). BAV is characterized by the presence of two asymmetrical aortic valve leaflets instead of three symmetrical ones (Henderson et al., 2022). BAV disrupts normal aortic valve flow patterns, resulting in abnormal turbulence and increased tissue stress (Ladich et al., 2011). Patients with BAV often develop premature calcific aortic valve disease, leading to valve stenosis and aortic valve insufficiency that requires valve replacement (Della Corte et al., 2007; Prakash et al., 2014; Roth et al., 2020; Tastet et al., 2017; Yuan and Jing, 2010).

The genetic architecture of BAV is complex, with low-penetrance variants, variable clinical expressivity and locus heterogeneity (Giusti et al., 2017), suggesting the involvement of multiple gene–gene and gene–environment interactions. Genome-wide association studies have identified several chromosomal regions, such as 18q, which harbors the gene encoding the GATA6 transcription factor, among several genes associated with BAV (Martin et al., 2007). In mice, *Gata6* is expressed in the myocardium, endocardium, cardiac neural crest and vascular smooth muscle (Morrisey et al., 1996), suggesting diverse functions in heart morphogenesis that remain to be fully elucidated*.*

*GATA6* variants lead to a variety of cardiac phenotypes ranging from structural malformations to conduction defects (Lepore et al., 2006; Škorić-Milosavljević et al., 2019). *GATA6* variants have been associated with familial BAV (Xu et al., 2018), but have been mostly found in sporadic cases (Alonso-Montes et al., 2018; Ma et al., 2021). The phenotypes of mice lacking *Gata6* highlight essential roles in organismal development. *Gata6*-null mice are early [embryonic day (E) 5.5] embryonic lethal due to extra-embryonic defects (Morrisey et al., 1998), and haploinsufficiency results in 60% right and left leaflet fusion (RL)-type BAV (Gharibeh et al., 2018). GATA6 is required for smooth muscle cell (SMC) differentiation and plays a crucial role in the patterning of the aortic arch arteries (Lepore et al., 2006). However, the underlying cellular and molecular mechanisms leading to BAV are still poorly defined.

In this study, we sought to elucidate the cellular and molecular pathways that underlie the function of GATA6 in aortic valve formation. We identified cellular defects that contribute to aberrant aortic valve morphogenesis and show that GATA6 regulates molecular programs directing cell migratory and invasive processes required for endocardial cushion development. Specifically, we demonstrate that GATA6 is necessary for the pro-migratory and invasive functions of C-X-C chemokine receptor type 7 (CXCR7; also known as atypical chemokine receptor 3 or ACKR3), a key regulator of chemokine signaling.

## RESULTS

### *Gata6*-null heterozygous mice show BAV and severe aortic insufficiency

We targeted exon 2 of the *Gata6* gene, encoding the large GATA-N domain of the protein (Fig. S1A). Indels introduced by CRISPR-Cas9 editing led to a frameshift mutation giving rise to a premature termination codon at amino acid position 291 (V291X), designated henceforth as *Gata6^STOP/+^* (Fig. S1A,B)*.* Histological analysis of E16.5 *Gata6^STOP/+^* mice revealed a right-non-coronary (RN)-type BAV morphology, based on coronary ostia positions relative to the aortic valve leaflets (Fig. S1C), with nearly 70% (20 of 30) penetrance, and a peri-membranous ventricular septal defect (VSD) with 43% (13 of 30) penetrance (Fig. S1D).

**Fig. 1.**
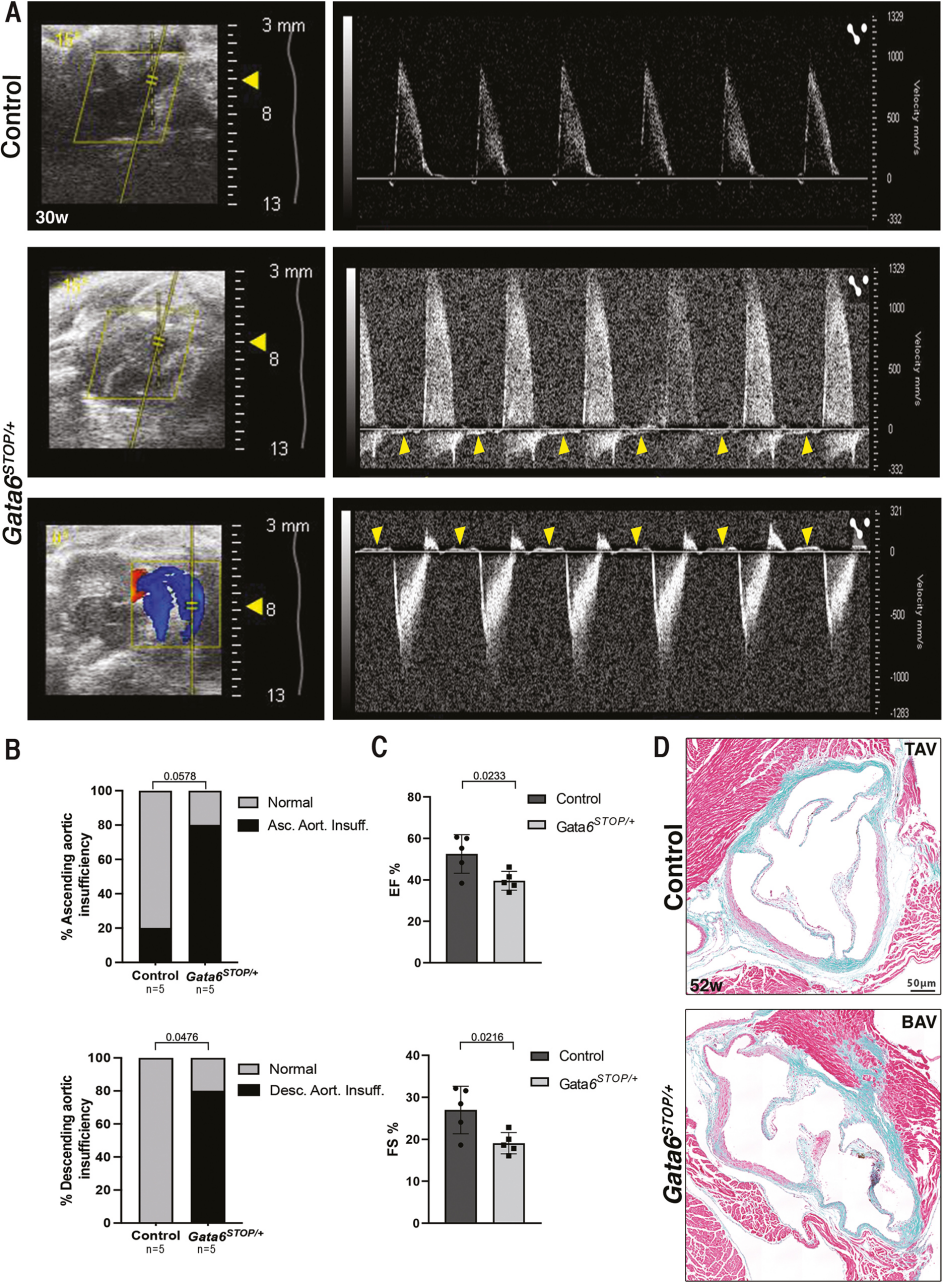
***Gata6^STOP/+^* mice have severe aortic insufficiency and systolic dysfunction.** (A) Echocardiographic images of 30±4 week control and *Gata6^STOP/+^* mice hearts. Yellow boxes represent the acoustic windows. Top: normal blood flow of ascending aorta. Middle and bottom: regurgitation in ascending and descending aorta in *Gata6^STOP/+^* mice, respectively. Blue and red coloring represent the pulsed wave Doppler capture. Yellow arrowheads indicate retrograde flow during diastole. *n*=5 mice. (B) Quantification of ascending and descending aortic insufficiency. *P*-values were obtained by Fisher's Exact test. (C) Quantification of the percentages of left ventricular ejection fraction (EF) and fractional shortening (FS). Data are represented as mean±s.d. *P*-values were obtained by unpaired two-tailed Student's *t*-test. *n*=5 mice. (D) Movat's pentachromic staining in 52-week-old control and *Gata6^STOP/+^* mice. *n*=5 mice. BAV, bicuspid aortic valve; TAV, tricuspid aortic valve. Scale bar: 50 μm.

To determine the effect of *Gata6* haploinsufficiency on cardiac function, we examined 30-week-old adult mice by spectral Doppler echocardiography of the ascending and descending aorta (Fig. 1A). Severe aortic regurgitation was observed in the ascending and especially the descending aorta in 80% (4 of 5) of *Gata6^STOP/+^* mice (Fig. 1A,B), indicating aortic insufficiency due to valve dysfunction. Moreover, ejection fraction and fractional shortening were also reduced in *Gata6^STOP/+^* mice (Fig. 1C), suggesting systolic dysfunction. Other parameters, i.e. left ventricular (LV) diastolic volume, LV systolic volume and LV mass, as well as flow velocity across the mitral valve and strain (percentage of aortic deformation), were comparable between the *Gata6^STOP/+^* mutant and control mice (Fig. S2). Movat's staining (Garvey et al., 1986) in 52-week-old mice aortic valve sections did not show any obvious differences in collagen deposition, elastin fibers or muscle mucin or fibrin composition (Fig. 1D).

### BAV associates with aberrant OFT development in *Gata6* haploinsufficient mice

**Fig. 2.**
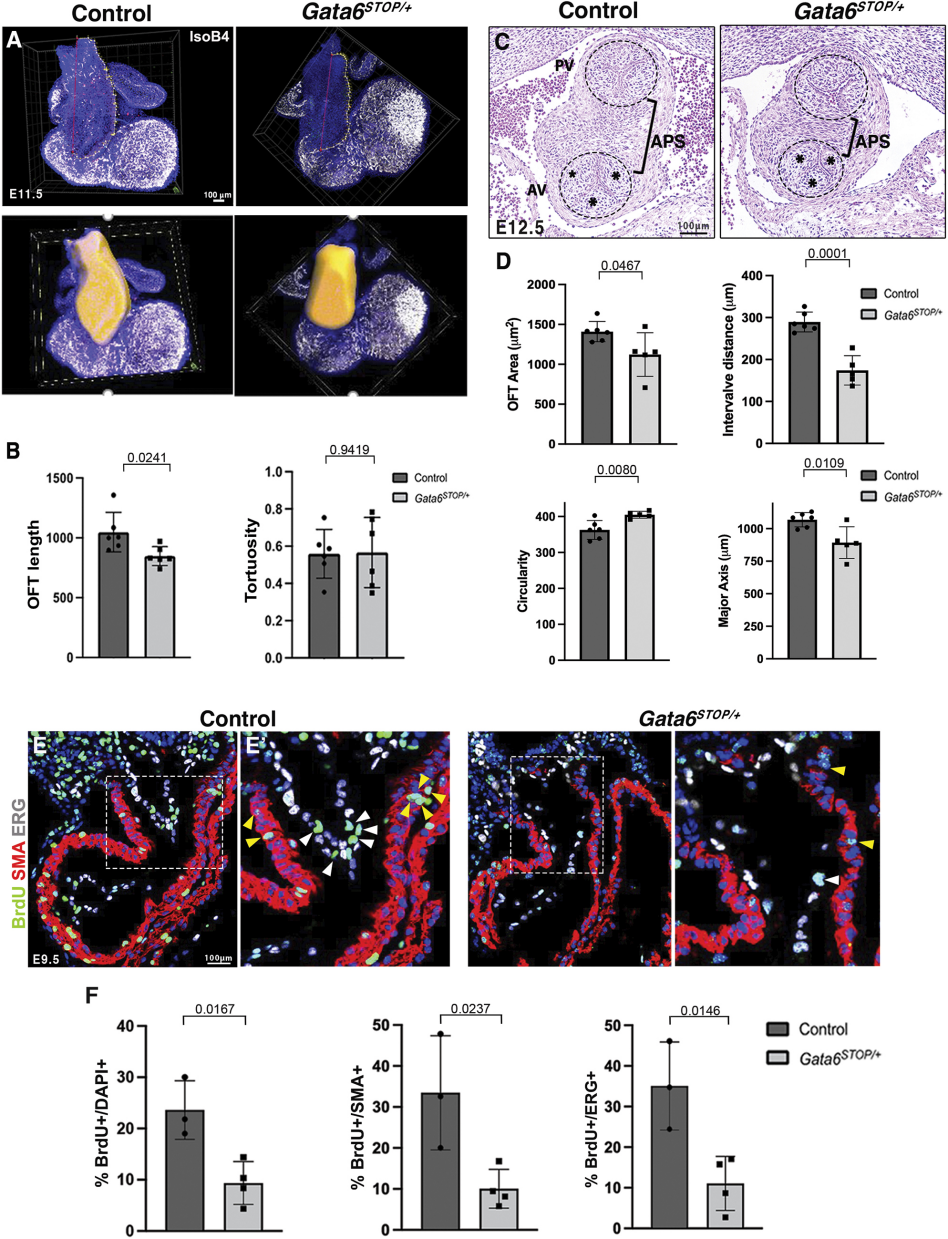
**Outflow tracts of *Gata6^STOP/+^* mice are shorter and narrower, which is associated with reduced proliferation.** (A) Whole-heart immunostaining images of E11.5 *Gata6^STOP/+^* and control mice. Top: the red line indicates outflow tract (OFT) linear length. The yellow line indicates length considering OFT tortuosity. Isolectin B4 (IsoB4, white) was used to label the endocardium with DAPI counterstaining (blue). Bottom: 3D OFT modeling with IMARIS software. The OFT region is rendered in yellow. Scale bar: 100 µm. (B) Quantification of OFT linear length and tortuosity measurements. Data are represented as mean±s.d. *P*-values were obtained by unpaired two-tailed Student's *t*-test. (C) Hematoxylin and Eosin staining of OFT frontal sections of E12.5 *Gata6^STOP/+^* and control mice. Asterisks indicate the position of the leaflets. Brackets indicate the aorticopulmonary septum (APS). Dashed circles delimit the aortic valve (AV) and pulmonary valve (PV). (D) Quantification of OFT area, inter-valve distance, circularity and major axis measured using Fiji Image software. Data are represented as means±s.d. *P*-values were obtained by unpaired two-tailed Student's *t*-test. *n*=6 control and 5 *Gata6^STOP/+^* embryos. (E) BrdU immunostaining of E9.5 *Gata6^STOP/+^* and control embryos. BrdU labels proliferating cells (green), α-SMA demarcates the myocardium (red), ERG demarcates endocardial cell nuclei (white), and nuclear counterstaining with DAPI is shown (blue). (E′) High magnification views of the boxed areas. Yellow arrowheads indicate BrdU^+^ myocardial cells, and white arrowheads indicate BrdU^+^ endocardial cells. (F) Quantification of the percentage of BrdU^+^ cells to DAPI^+^, SMA^+^ and ERG^+^ cells in the OFT. Data are represented as mean±s.d. *P*-values were obtained by unpaired two-tailed Student's *t*-test. *n*=3 control and 4 *Gata6^STOP/+^* embryos.

We performed isolectin B4 (IsoB4) whole-mount immunostaining of the ventricular endocardium and blood vessel endothelial cells, followed by IMARIS three-dimensional (3D) modeling and volume rendering to determine OFT morphology at E11.5 (Fig. 2A, Movies 1 and 2). E11.5 *Gata6^STOP/+^* mice displayed a shorter OFT compared to that in littermate controls (Fig. 2B). Moreover, we measured tortuosity as a parameter of OFT curvature and found it to be unchanged (Fig. 2B). We also examined OFT caliber as it relates to the separation of the aortic and pulmonary valves by the aorticopulmonary septum (APS), which occurs between E11.5 and E12.5 (Fernández et al., 2009) (Fig. 2C). OFT dimensions in E12.5 *Gata6^STOP/+^* mice were 20% below normal and led to narrowing of the OFT caliber (Fig. 2D). The shortened distance between primitive pulmonary and aortic valves (Fig. 2D) reduced the APS and gave rise to a more circular OFT (Fig. 2D). The length of the major (ventral-dorsal) axis of the OFT was also significantly reduced (Fig. 2D).

To further define the cellular mechanism causing OFT maldevelopment in *Gata6^STOP/+^* mice, we examined cell proliferation in E9.5 hearts, specifically in the S phase of the cell cycle by bromodeoxyuridine (BrdU) immunostaining (Fig. 2E,E′). Proliferation was reduced in *Gata6^STOP/+^* OFT (Fig. 2E,E′), in both the myocardium and endocardium (Fig. 2F). These findings indicate that the shortened and narrowed OFT in *Gata6^STOP/+^* mice can be ascribed to decreased myocardial and endocardial cell proliferation.

### Cell-autonomous requirement for GATA6 in the second heart field

We next sought to establish GATA6 requirement in cardiovascular development (Kisanuki et al., 2001). Endothelial-specific *Gata6* deletion using the *Tie2^Cre^* driver line resulted in overriding aorta with 45% (5 of 11) penetrance (Fig. S3A,B). *Gata6* deletion in the myocardium, endocardium (Stanley et al., 2002) and epicardium (Zhou et al., 2008) using the *Nkx2.5^Cre^* driver line caused fully penetrant VSD (Fig. S3C,D) and was lethal at birth (Table S1) (van Berlo et al., 2010). In addition, *Gata6^flox/flox^*; *Nkx2.5^Cre^* mutants showed extensive hypertrabeculation and a thinner compact ventricular wall, consistent with the key roles of GATA6 in the endocardium and myocardium during ventricular chamber development (van Berlo et al., 2010). None of these endocardial- or endothelial-specific models recapitulated the BAV phenotype (Fig. S3B,D).

**Fig. 3.**
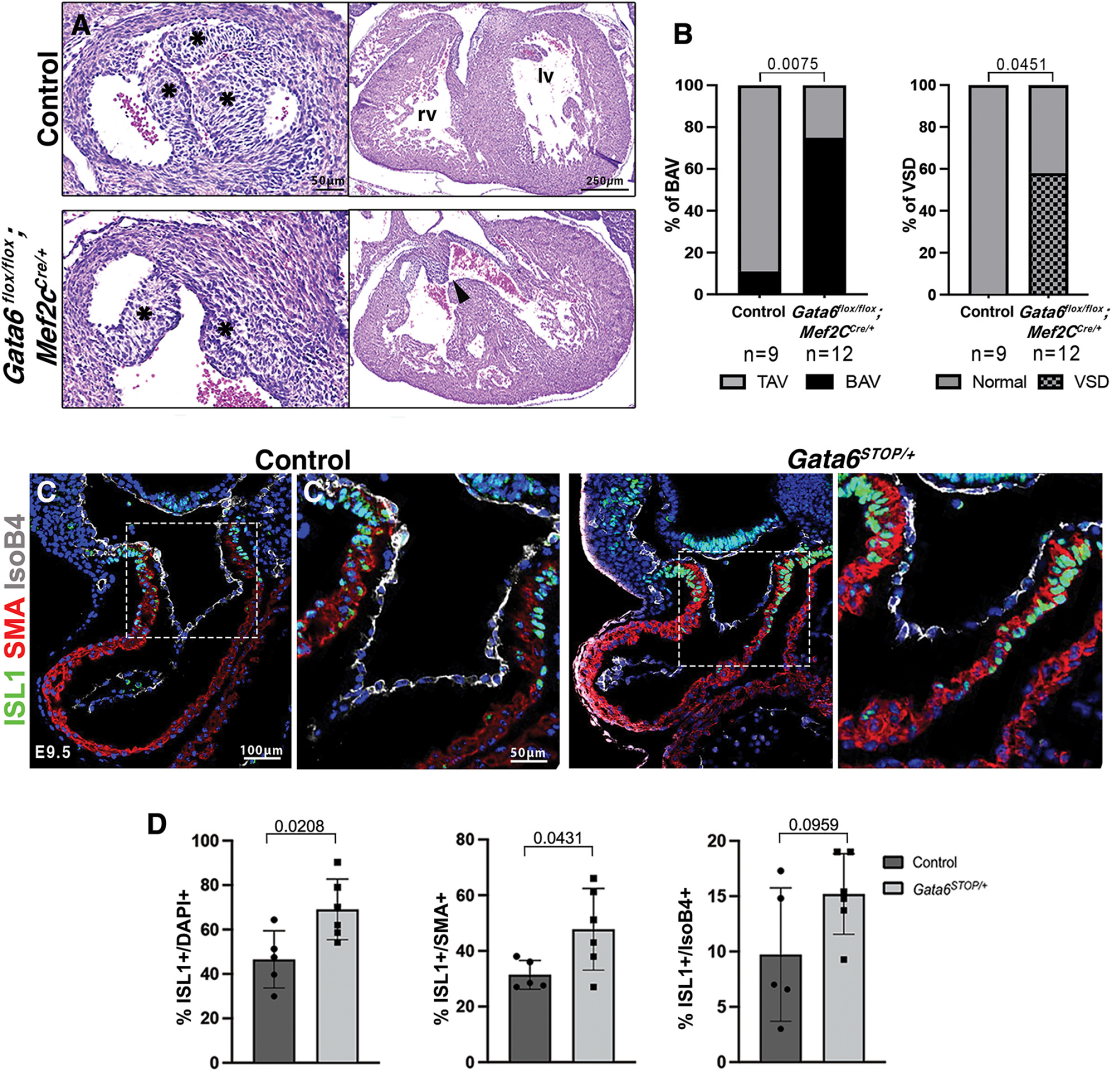
**Cell-autonomous *Gata6* requirement in the secondary heart field.** (A) Hematoxylin and Eosin staining of sections of the aortic valve (right) and ventricles (left) from E16.5 *Gata6^flox/flox^;Mef2c^Cre^* and control mice. Asterisks indicate the position of the leaflets. The black arrowhead indicates ventricular septal defect (VSD). lv, left ventricle; rv, right ventricle. (B) Quantification of the percentage of embryos with bicuspid aortic valve (BAV) and VSD. *P*-values were obtained by Fisher exact test. *n*=9 control and 12 *Gata6^flox/flox^;Mef2c^Cre^* embryos. TAV, tricuspid aortic valve. (C) Fluorescence immunostaining on E9.5 *Gata6^STOP/+^* and control OFT sections. ISL1 labeling marks secondary heart field progenitors (green), αSMA demarcates the myocardium (red), IsoB4 demarcates the endocardium (white), and nuclear counterstaining with DAPI is shown (blue). (C′) Higher magnification views of the boxed areas. (D) Quantification of the percentage of ISL1^+^ cells to total, SMA^+^ and IsoB4^+^ cells in the OFT. Data are represented as mean±s.d. *P*-values were obtained by unpaired two-tailed Student's *t*-test. *n*=5 control and 6 *Gata6^STOP/+^* embryos.

To determine GATA6 requirement in early cardiac progenitors, we used the *Mef2c^Cre^* driver line, active in anterior or secondary heart field (SHF)-derived endothelial and myocardial progenitors that give rise to the OFT, right ventricle and ventricular septum (Verzi et al., 2005). *Gata6^flox/flox^*; *Mef2c^Cre^* mutants fully recapitulated the *Gata6^STOP/+^* BAV and VSD phenotypes (Fig. 3A), with 75% (9 of 12) and 58% (7 of 12) penetrance, respectively (Fig. 3B). These data suggest that GATA6 is required cell-autonomously in SHF-derived progenitors.

To gain insight, we examined the SHF marker islet-1 (ISL1) (Quaranta et al., 2018) in *Gata6^STOP/+^* mice. ISL1 marks progenitors for cardiomyocytes, SMCs and endothelial cell lineages, and is gradually switched off as progenitors incorporate into the arterial pole of the heart between E8.5 and E10.5 (Bu et al., 2009). ISL1-positive immunostaining was significantly increased in the distal OFT myocardium, and marginally in the endocardium of E9.5 *Gata6^STOP/+^* hearts (Fig. 3C,D), suggesting that SHF-progenitor differentiation is deficient, whereas no differences were detected in pharyngeal mesoderm (Fig. S3E). These data indicate that defective OFT development in *Gata6^STOP/+^* mice can be ascribed to impaired SHF-derived progenitor differentiation.

### Deficient cNCC contribution and SMC differentiation in *Gata6^STOP/+^* OFT

**Fig. 4.**
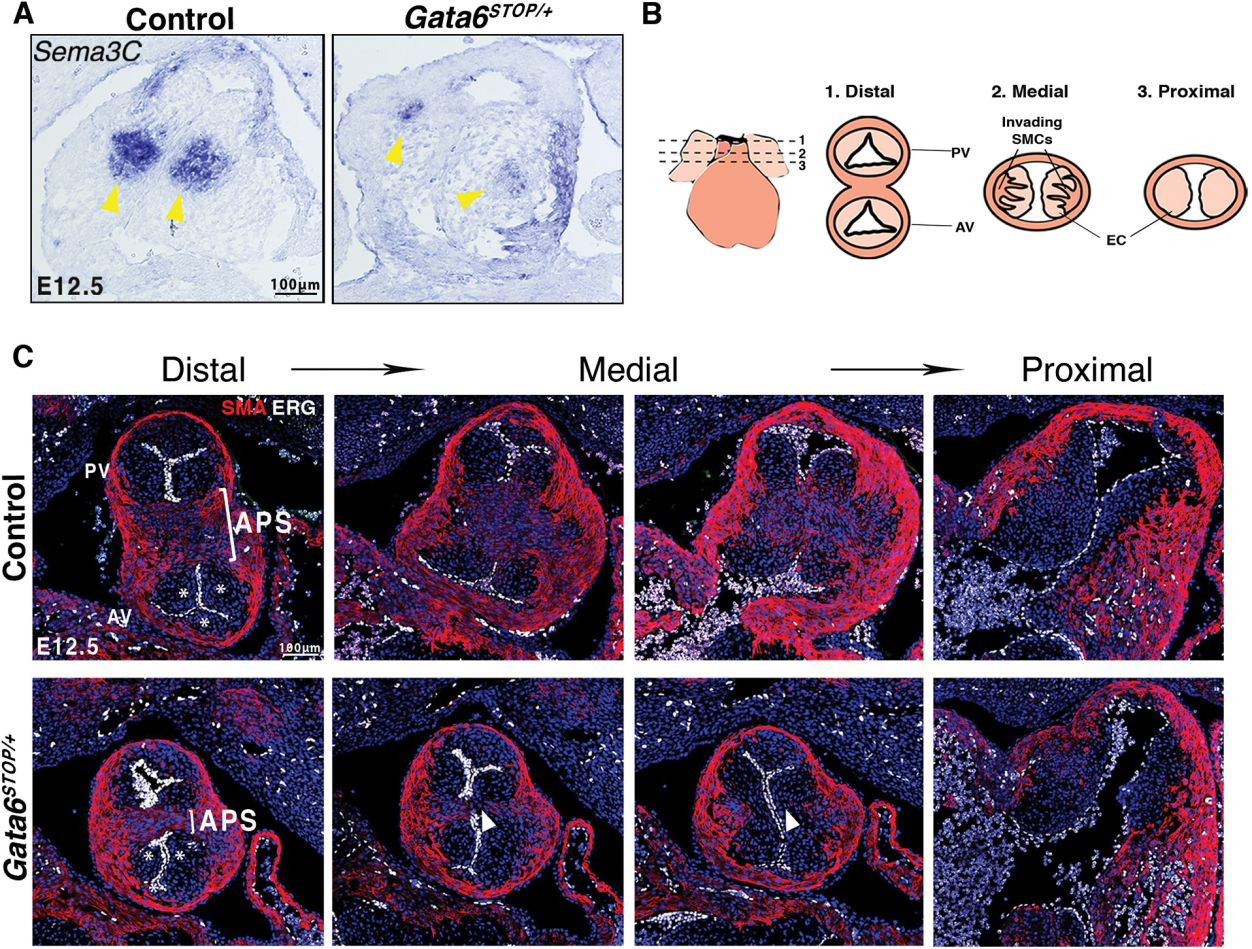
**Reduced contribution of cardiac neural crest cells to OFT septation in *Gata6^STOP/+^* mice**. (A) *In situ* hybridization of *Sema3c* in E12.5 control and *Gata6^STOP/+^* OFT sections. Yellow arrowheads indicate the absence of post-migratory cardiac neural crest cells in the mutant OFT. *n*=3 mice. (B) Schematic of distal to proximal OFT sectioning. AV, aortic valve; PV, pulmonary valve; SMC, smooth muscle cell, EC, endocardial cushion. (C) Fluorescence immunostaining for α-SMA (red) for myocardium and smooth muscle, and ERG (white) for endocardial cell nuclei, and nuclear counterstaining with DAPI (blue) in E12.5 *Gata6^STOP/+^* and control OFT sections. Asterisks indicate the position of the leaflets. White brackets indicate the aorticopulmonary septum (APS). White arrowheads indicate narrowed APS. *n*=3 mice.

cNCCs constitute a major reservoir of mesenchyme progenitors that are required for patterning of the endocardial cushions in the OFT and aortic arch arteries (Erhardt et al., 2021; Jain et al., 2011). We examined the expression of *Sema3c* (Fig. 4A), a marker of post-migratory cells and transcriptionally regulated by GATA6 (Brown et al., 2001; Kodo et al., 2009; Lepore et al., 2006). *In situ* hybridization on E12.5 *Gata6^STOP/+^* OFT frontal sections revealed markedly reduced *Sema3c* staining, suggesting reduced cNCC presence (Fig. 4A). Moreover, GATA6 is required for neural crest cell-derived SMC differentiation (Lepore et al., 2006; Losa et al., 2017). Analysis of smooth muscle differentiation along distal, medial and proximal segments of E12.5 *Gata6^STOP/+^* mice OFT (Fig. 4B) revealed deficient smooth muscle actin (SMA, encoded by *Acta2*)-positive immunostaining in the APS, consistent with reduced cNCC presence and/or impaired SMC differentiation (Fig. 4C)*.*

### *Gata6* regulates a pro-migratory gene signature in OFT development

**Fig. 5.**
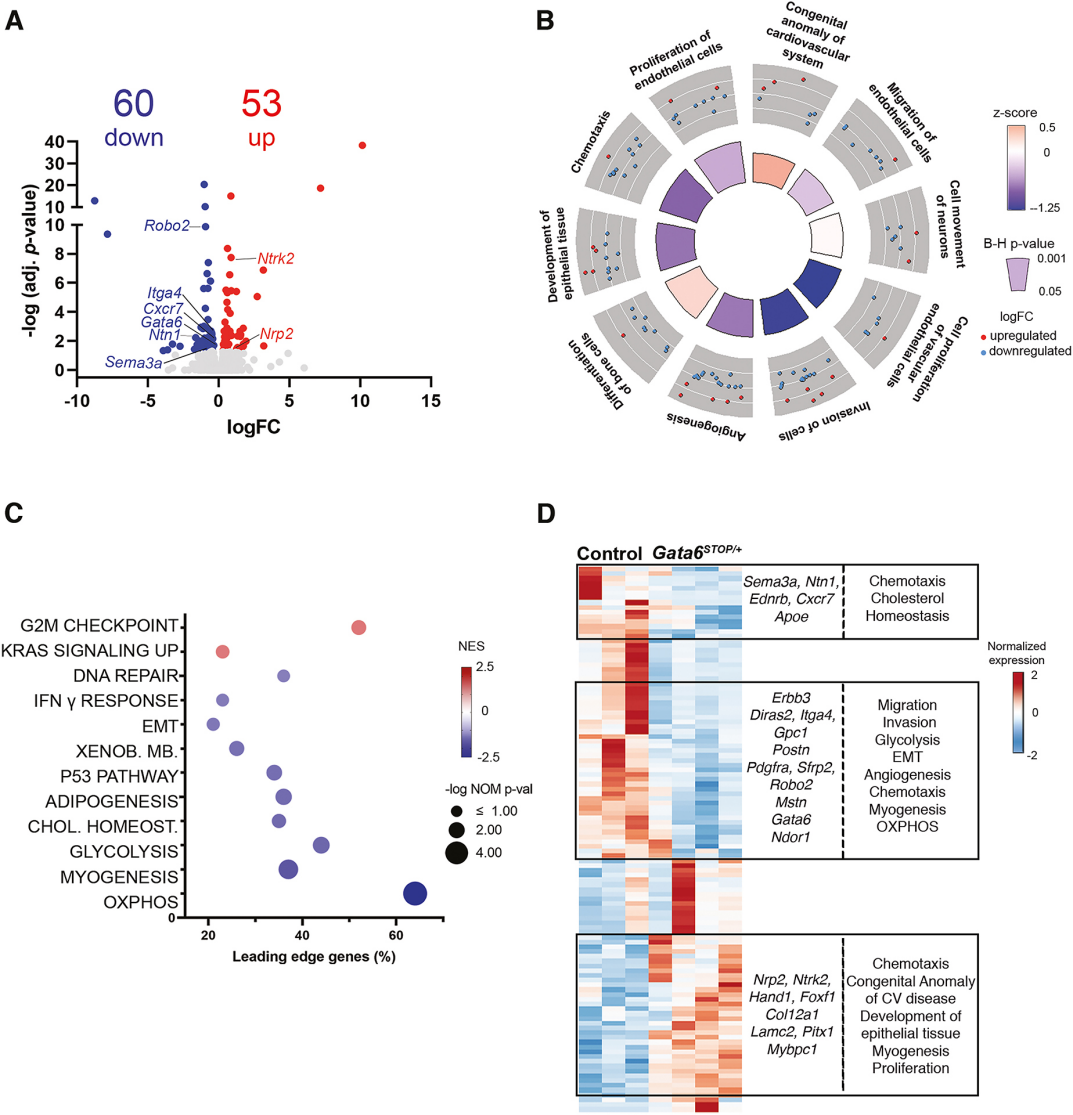
**Depleted cellular motility processes in E11.5 *Gata6^STOP/+^* OFT.** (A) Volcano plot of the genes detected in the RNA-seq analysis. Relevant differentially regulated genes are indicated. Significantly downregulated and upregulated genes (adjusted *P-*value <0.05) are labelled in blue and red, respectively. Non-differentially expressed genes are labelled in grey. FC, fold change. (B) The circle plot highlights ten Ingenuity Pathway Analysis disease and function terms enriched in *Gata6^STOP/+^* mice. Red and blue dots indicate upregulated and downregulated genes in the pathway, respectively. The heights of the inner circle sections are associated with Benjamini–Hochberg (B-H) *P*-values <0.05 (taller, more significant), and enrichment z-scores values are color coded from positive (orange) to negative (blue). (C) The bubble plot shows 12 enriched Hallmark gene sets by gene set enrichment analysis (*y*-axis). The negative logarithm of the nominal (NOM) *P-*value <0.1 is represented by the size of the bubble (bigger, more significant). Normalized enrichment scores (NES) are color coded from positive (red) to negative (blue). The percentage of leading edge genes is represented in the *x*-axis. OXPHOS, oxidative phosphorylation; XENOB. MB., xenobiotic metabolism. (D) Heatmap representation of the differentially expressed genes for each of the samples analyzed (adjusted *P-*value <0.05) of E11.5 *Gata6^STOP/+^* OFT versus controls. The expression intensity is plotted on a scale from red (upregulated) to blue (downregulated). CV, cardiovascular; EMT, epithelial-mesenchymal transition.

For further insight, we performed RNA sequencing (RNA-seq) on E11.5 *Gata6^STOP/+^* and control OFTs (Fig. 5). EdgeR identified 113 differentially expressed genes, of which 53 were upregulated and 60 downregulated (Fig. 5A; Table S2). As expected, *Gata6* expression was decreased by RNA-seq [log_2_(fold change or FC)=−0.42331; adjusted *P*-value=0.022647]. Quantitative real-time PCR confirmed that *Gata6* was expressed at 60% of the wild-type level (Fig. S3F). Ingenuity Pathway Analysis uncovered only a few enriched categories, i.e. ‘congenital anomaly of cardiovascular system’ (*Hand1*, *Foxf1*) and ‘differentiation of bone cells’ (*Postn*) (Fig. 5B,D). To expand the gene categories, we performed gene set enrichment analysis (GSEA) against ‘Hallmark’ sets (Liberzon et al., 2015) (Fig. 5C). Enriched pathways were ‘G2M checkpoint’ and ‘KRAS signaling UP’, potentially highlighting a proliferative defect.

In contrast, *Gata6^STOP/+^* OFT gene expression was depleted for functional categories such as ‘cell movement of neurons’ (*Cxcr7*, *Erbb3*), ‘chemotaxis’ (*Sema3a*, *Robo2*, *Ntn1*, *Nrp2*, *Ntrk2*), ‘invasion of cells’ (*Diras2*, *Itga4*) and ‘proliferation of endothelial cells’ (*Anxa8*) (Fig. 5B,D). Related categories such as ‘angiogenesis’ (*Pdgfra*, *Sfrp2*) and ‘development of epithelial tissue’ (*Col12a1*, *Lamc2*) were also depleted (Fig. 5B,D). Consistent with this, GSEA revealed depleted gene sets such as ‘epithelial-mesenchymal transition’ (Fig. 5C) (von Gise and Pu, 2012) and those related to cellular stress pathways such as ‘DNA repair’ and ‘P53 pathway’, which is potentially linked to ‘oxidative phosphorylation’ (*Ndor1*), ‘glycolysis’ (*Gpc1*) and lipid metabolism dysfunction [i.e. ‘cholesterol homeostasis’ (*Apoe*) and ‘adipogenesis’] (Fig. 5C,D). Moreover, depleted ‘myogenesis’ (*Mstn*, *Mybpc1*) is consistent with GATA6 requirement for myocardial differentiation (Sharma et al., 2020).

Altogether, the bioinformatic analysis indicates that GATA6 regulates a set of processes associated with cellular proliferation and especially migration, potentially involved in development and patterning of the OFT.

### CXCR7 mediates GATA6-dependent migration and invasion

Endocardial cushion explant assays can recapitulate morphogenetic processes occurring *in vivo*, including the epithelial-mesenchymal transition and proliferation (Runyan and Markwald, 1983; Timmerman et al., 2004). Therefore, to corroborate the potential link between OFT underdevelopment and defective cell migration in *Gata6^STOP/+^* mutants, we performed explant assays (Fig. S4A). SMA^+^ mesenchymal cells migrating out from OFT explants are derived from both the endocardial-mesenchymal transition and neural crest cells that have colonized the endocardial cushions (MacGrogan et al., 2016; Ridge et al., 2021). Consistent with the assessment in the RNA-seq analysis, mesenchymal migratory and invasive behaviors in E11.5 *Gata6^STOP/+^* explants were below normal, whereas proliferation was unchanged (Fig. S4B,C).

We then focused on *Cxcr7* as a potential mediator of *Gata6* function in the OFT. *Cxcr7* gene expression was found to be decreased in *Gata6^STOP/+^* OFT (FC=−1.27, adjusted *P*-value=0.0196) (Fig. 5A; Table S2) and, importantly, *Cxcr7* mutant mice recapitulate valvulo-septal phenotypes found in the *Gata6^STOP/+^* mutants (Sierro et al., 2007). CXCR7, together with the chemokine receptor CXCR4 and its ligand CXCL12 (also known as SDF1), regulates cellular migration and proliferation in many developmental and pathological settings (Döring et al., 2014).

We evaluated CXCR7 requirement for GATA6-mediated cellular motility*.* We tested the effect of VUF11207, a specific CXCR7 agonist that induces recruitment of the cytosolic scaffolding protein β-arrestin, followed by its internalization and downstream pathway activation (Wijtmans et al., 2012). Thus, whereas supplementing wild-type OFT explants with 100 nM VUF11207 had no effect (Fig. S5A,A′,B,B′,D), supplementing with 1 µM VUF11207 led to a significant dose-dependent decline in migration, defined as the furthest distance migrated (Fig. S5A,A′,C,Cʹ,D). In contrast, a dose-dependent increase was found with 1 µM of VUF11207 for the invasion parameter, defined as the furthest distance invaded, relative to that seen in the control (Fig. S5A″,C″,D), whereas supplementing with 100 nM VUF11207 had no effect (Fig. S5A″,B″,D). However, providing the explants with either 100 nM or 1 µM of the agonist did not result in any proliferative changes (Fig. S5D).

**Fig. 6.**
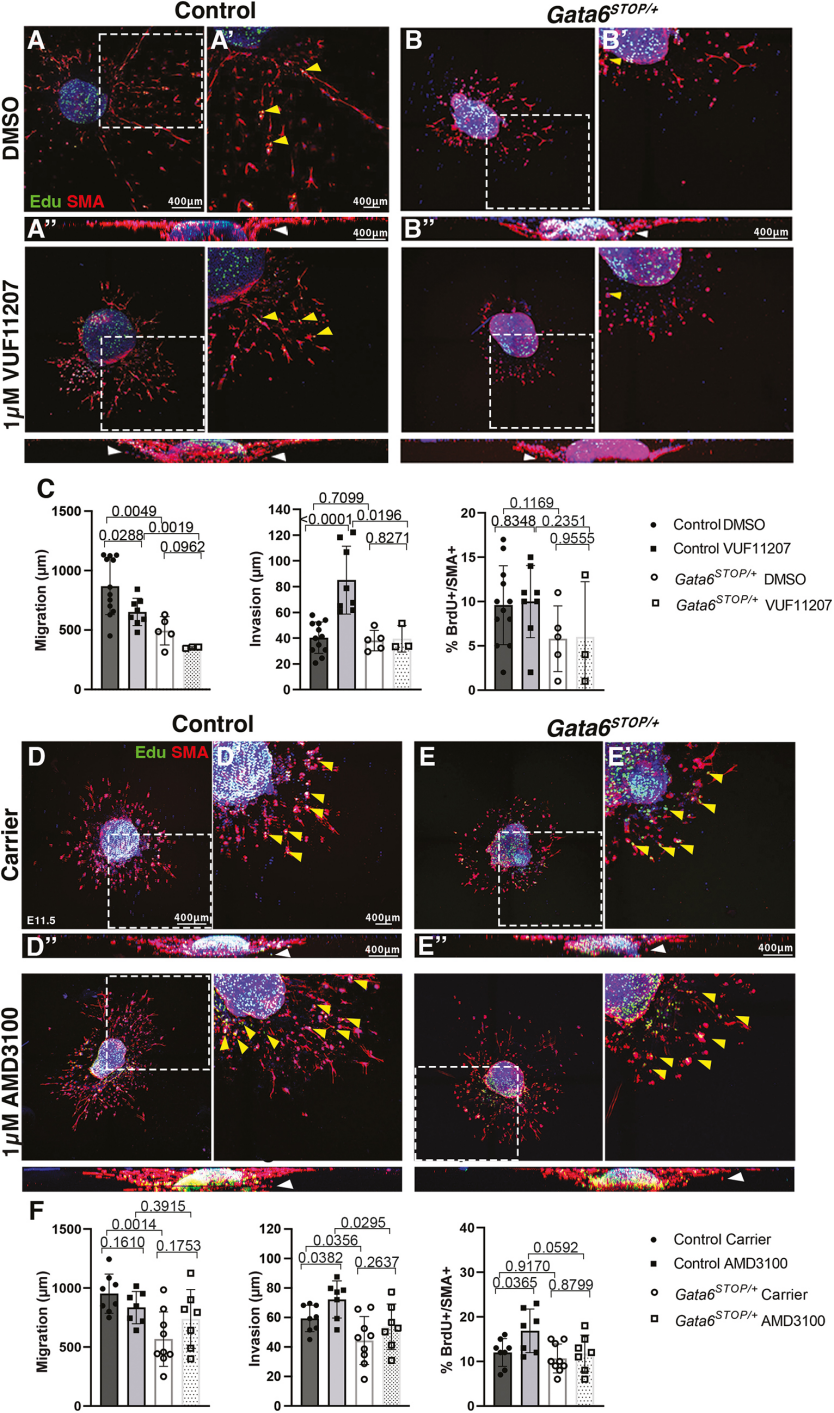
**CXCR7 mediates GATA6 regulation of mesenchymal cell migration.** (A,B,D,E) E11.5 control and *Gata6^STOP/+^* explants supplemented with 1 μM VUF11207 (A,B) or 1 μM AMD3100 (D,E). (A′,B′,Dʹ,Eʹ) Magnification of the boxed areas in A,B,D,E of the body of the explant and outwardly migrating mesenchymal cells. (A″,B″,D″,E″) Two-dimensional orthogonal views of the explants showing mesenchymal cell invasion into the collagen gel. White arrowheads indicate invading cells. Proliferating cells (green) are indicated by yellow arrowheads. α-SMA demarcates the mesenchyme (red). Nuclear counterstaining with DAPI is shown (blue). (C,F) Quantification of mesenchymal migration, invasion and proliferation following treatment with 1 μM VUF11207 (C) or 1 μM AMD3100 (F). Data are represented as mean±s.d. *P*-values were obtained by unpaired two-tailed Student's *t*-test. For C, *n*=12 control DMSO, 8 control VUF11207, 5 *Gata6^STOP/+^* DMSO, 3 *Gata6^STOP/+^* VUF11207. For F, *n*=8 control carrier, 7 control AMD3100, 9 *Gata6^STOP/+^* carrier, 7 *Gata6^STOP/+^* AMD3100.

Next, consistent with our pilot experiment, supplementing control explants with 1 µM VUF11207 resulted in decreased migration (Fig. 6A,A′,C) and increased invasion (Fig. 6A,A″,C), confirming that migratory processes taking place in endocardial cushion development are regulated by CXCR7. However, *Gata6^STOP/+^* explants did not respond to 1 µM VUF11207 supplementation (Fig. 6B,B″,C), suggesting that migration and invasion mediated by CXCR7 are dependent on *Gata6*. No effects on proliferation were observed in either *Gata6^STOP/+^* or littermate control explants (Fig. 6C).

CXCR7 modulates CXCR4 function by sequestering CXCL12 (Boldajipour et al., 2008; Chen et al., 2022; Naumann et al., 2010). To determine whether CXCR7 modulation of CXCR4 is dependent on *Gata6*, we tested the effects of AMD3100, a dual-function chemical compound known to simultaneously antagonize CXCR4 (Debnath et al., 2013) and allosterically activate CXCR7 (Kalatskaya et al., 2009). Having previously defined an optimal dose-dependent AMD3100 concentration (Ferreira Oliveira, 2020), we found that supplementing wild-type OFT explants with 1 µM AMD3100 did not affect migration (Fig. 6D,D′,F) but significantly increased invasion (Fig. 6D,D″,F) and proliferation (Fig. 6D,D′,F). This suggests that CXCR4 regulates mesenchymal cell proliferation in OFT explants, whereas CXCR7 likely regulates migration and invasion. Importantly, supplementing *Gata6^STOP/+^* OFT explants with 1 µM AMD3100 did not alter mesenchymal cell migration, invasion or proliferation relative to that in *Gata6^STOP/+^* OFT explants in the absence of AMD3100 (Fig. 6E,E″,F). Thus, *Gata6* is required for the pro-invasive effects mediated by simultaneous CXCR4 inhibition and CXCR7 activation via AMD3100, but not for pro-proliferative effects.

## DISCUSSION

In this study, we show that *Gata6* haploinsufficiency results in highly penetrant BAV and characterize the phenotype to OFT alignment anomalies, including a membranous VSD, a common component of congenital heart disease in humans. We elucidate the deficient cellular differentiation and proliferation mechanisms associated with congenital heart defects in *Gata6^STOP/+^* mice. We find a requirement for functional hierarchy between GATA6 and CXCR7 in mesenchymal cell migration and invasion in endocardial cushion development.

The failure to elongate the OFT likely underlies the ventricular-arterial alignment defects observed in *Gata6^STOP/+^* embryos. The OFT length in *Gata6^STOP/+^* embryos may be insufficient for normal ventricular septal alignment and atrioventricular canal endocardial cushion fusion (Gittenberger-de Groot et al., 2014). Normally, SHF progenitors are added progressively to the arterial pole, which subsequently becomes incorporated into the ventricular outlets at the base of the great arteries (Rochais et al., 2009). Deficient SHF deployment is the primary cause of septal defects at the ventricular-arterial connection with the OFT (Gittenberger-de Groot et al., 2014). The delayed differentiation of ISL1-positive progenitors and decreased proliferation of distal OFT endocardial/myocardial lineages observed in *Gata6^STOP/+^* mice are consistent with dysregulation in SHF-derived progenitors.

A cell-autonomous GATA6 requirement in the SHF is further supported by conditional *Gata6* inactivation, which reproduces the *Gata6^STOP/+^* mutant phenotypes. Moreover, interactions between the SHF and cNCCs are crucial for normal OFT development, and perturbed SHF signaling indirectly impacts cNCC distribution in the endocardial cushions (Ward et al., 2005), causing mispatterning of the aortic valve leaflets (Waldo et al., 2005). GATA6 is also required cell-autonomously in post-migratory cNCCs for *Sema3c* expression, smooth muscle differentiation and aortic arch artery patterning (Lepore et al., 2005). Medial arterial defects have been previously linked to BAV aortopathy (Yassine et al., 2017), and aortic structural integrity is likely compromised in the adult *Gata6^STOP/+^* mice with BAV, based on our spectral Doppler echocardiography experiments revealing retrograde flow (ascending and descending) and severe aortic regurgitation.

Cell movement and invasion are necessary for organogenesis (Aman and Piotrowski, 2010). GATA factors control processes that repress epithelial characteristics and confer migratory capabilities to epithelial cells involved in mesoderm development, neural crest formation and wound healing (Campbell et al., 2011). Consistent with this, our transcriptional profiling suggests that pro-migratory and invasive processes are globally impaired in *Gata6^STOP/+^* OFT development. These pathways principally involve canonical cell guidance molecules and receptors, including semaphorins, ephrins and netrins, and oncogenic pathways such as ERBB3 and NTRK. Mis-regulation of these pathways has been associated with developmental patterning defects and congenital heart disease (Claro and Ferro, 2020; Erickson et al., 1997; Hang et al., 2021; Mommersteeg et al., 2015).

We focused on *Cxcr7* as potentially acting downstream of GATA6. CXCR7 is a decoy receptor for CXCL12–CXCR4 signaling with broad effects on chemotaxis, cell proliferation and migration (Chédotal et al., 2005; Shi et al., 2020). Moreover, this chemokine pathway plays key role(s) in aortic valve development (Ivins et al., 2015; Ridge et al., 2021; Sierro et al., 2007; Yu et al., 2011) and its disruption recapitulates *Gata6^STOP/+^* phenotypes (Duval et al., 2022; Ivins et al., 2015; Sierro et al., 2007; Yu et al., 2011). As expected, we found that agonist-mediated activation of CXCR7 inhibited migration and increased invasion, likely by blocking the pro-migratory effects of CXCL12–CXCR4 signaling (Wijtmans et al., 2012). We also expected that blocking CXCL12 binding to CXCR4 might impair migration (Ridge et al., 2021; Yu et al., 2011) but, given that the antagonist simultaneously activated CXCR7 (Kalatskaya et al., 2009), only effects on invasion and proliferation were found. This anti-invasive and anti-proliferative response is in agreement with previous findings indicating that CXCL12–CXCR4 signaling promotes migratory processes and inhibits proliferation in the valve mesenchyme (Ridge et al., 2021). Regardless, even in the presence of highly potent CXCR7 activity modulators, no effects were found in *Gata6^STOP/+^* mutants, suggesting that GATA6 is required for CXCR7-mediated migratory or invasive processes in endocardial cushion formation. Our findings are summarized in Fig. S5E,F. Proliferation was not affected in the *Gata6^STOP/+^* explants or by the modulators of CXCR7 activity, suggesting that proliferation and migration/invasion may be independently regulated in this context. We have not found any bioinformatic evidence of GATA-binding sequences in proximity to the *Cxcr7* gene supportive of direct transcriptional regulation, suggesting that GATA6 control of *Cxcr7* could be indirect. Further research is required to understand the mechanisms by which GATA6 regulates CXCR7 and CXCL12–CXCR4 signaling during endocardial cushion formation and in congenital heart disease.

## MATERIALS AND METHODS

### Animal studies and generation of the *Gata6^STOP/+^* mouse line

Animal experiments adhered to Animal Research: Reporting of *In Vivo* Experiments (ARRIVE) guidelines and complied with ethical standards outlined in the UK Animal (Scientific Procedures) Act 1986, Royal Decree 53/2013, EU Directive 2010/63/EU and Recommendation 2007/526/EC on the protection of animals used for experimental and other scientific purposes, enacted in Spanish law under Real Decreto 1201/2005. Approval was obtained from the CNIC Animal Experimentation Ethics Committee, Complutense University of Madrid, and Community of Madrid (reference PROEX 155.7/20).

The standard knockout *Gata6^STOP^* mouse line was generated using CRISPR-Cas9 technology. We designed a specific CRISPR RNA (crRNA) using the Spanish National Biotechnology Centre (CNB)-Spanish National Research Council (CSIC) web tool Breaking-Cas (https://bioinfogp.cnb.csic.es/tools/breakingcas/) and CRISPOR-TEFOR online tool (http://crispor.gi.ucsc.edu/crispor.py). The sequences with higher specificity, fewer off-targets and higher efficiency scores were selected (Table S3). crRNA sequences were used as annealed two-part synthetic crRNA (Alt-RR CRISPR-Cas9 crRNA, 2 nmol, Integrated DNA Technologies or IDT) and trans-activating crRNA (tracrRNA; Alt-RR CRISPR-Cas9 tracrRNA, 5 nmol, IDT, 1072532) molecules, known as the Alt-R CRISPR-Cas9 system. We used the Alt-RR *Streptococcus pyogenes* Cas9 nuclease (IDT, 1081058). Single-stranded DNA templates were custom synthetic genes (Megamer single-stranded gene fragments, IDT). The complete guide RNA complex (annealed crRNA and tracrRNA) was diluted in microinjection buffer (1 mM Tris HCl, pH 7.5; 0.1 mM EDTA) and incubated with Cas9 protein to obtain active ribonucleoprotein complexes. The final concentrations of components in ribonucleoprotein preparations were 0.15-0.61 pmol/μl of crRNA and tracrRNA, 20-30 ng/μl of Cas9 protein and 10 ng/μl of single-stranded oligodeoxynucleotides (Table S3). The final injection mixes were passed through centrifugal filter units (UFC30VV25, EMD Millipore) and spun at 21,000 ***g*** for 5 min at room temperature. Reagents were microinjected into one-cell fertilized C57BL/6 mouse embryos (Harms et al., 2014). Pups were screened for the targeted mutations using external primers by PCR (Table S3). Sanger sequencing was performed to confirm target gene editing. The selected founder was backcrossed to the C57BL/6 background.

Other mouse strains used in this study are as follows: *Gata6^flox^* (Sodhi et al., 2006), *Tie2^Cre^* (Kisanuki et al., 2001), *Nkx2.5^Cre^* (Stanley et al., 2002) and *Mef2c^Cre^* (Verzi et al., 2005).

### Tissue processing for histological procedures

For histological procedures, whole embryos or torsos were fixed in 4% paraformaldehyde (PFA, Electron Microscopy Sciences, 50980487) overnight at 4°C. Paraffin-embedded embryos/torsos were cut into 7 μm sections using a HistoCore AUTOCUT rotary microtome (Leica Biosystems). Hematoxylin and Eosin staining was performed according to standard protocols.

### RNA probe synthesis and *in situ* hybridization

Antisense RNA probes were designed to be exon spanning or complementary to the 3′ untranslated region sequences. Targeted regions were amplified by PCR using 100 ng of cDNA from hearts. The PCR product was ligated to the pGEM-T easy vector (Promega, A1360) and transformed into DH5α competent *Escherichia coli* cells. Plasmid DNA was isolated from positive colonies, linearized and transcribed by T7 or Sp6 polymerase (RPOLT7-RO and RPOLSP6-RO, Roche). The RNA probes were purified and stored at −80°C. Primer sequences used for probe generation are listed in Table S3. *In situ* hybridization was performed as described previously (Kanzler et al., 1998).

### Immunohistochemistry

Paraffin-embedded 7 μm sections were citrate-unmasked and incubated overnight with the primary antibodies. For antigens that did not require amplification, fluorescent dye-conjugated secondary antibodies were directly incubated for 1 h. For signal amplification, a secondary biotin-conjugated antibody was used, and signal detection was performed using the Tyramide Signal Amplification Plus Fluorescein kit (PerkinElmer, NEL741B001KT). All antibodies used are listed in Table S3.

### Whole-mount immunofluorescence

E11.5 embryos were fixed in 4% PFA overnight at 4°C and then kept in PBS at 4°C. Tissue clearing was performed with CUBIC-1 (Susaki et al., 2014) at 37°C for 1.5 days. Embryos were incubated with primary antibody endomucin (V.7C7) (Santa Cruz Biotechnology, sc-65495) at 4°C for 3 days. Incubation with secondary antibody was performed at 4°C for 3 days. Then, incubation with CUBIC-2 (Susaki et al., 2014) was performed at room temperature overnight. Quantification of OFT length and tortuosity and 3D modeling were performed with IMARIS software. Six embryos of each genotype were studied. Statistical analyses were performed using unpaired two-tailed Student's *t*-test, and differences were considered statistically significant at *P*<0.05. Data are presented as mean±s.d.

### OFT explant assay

Collagen gels were prepared by pouring a solution of 1.5 mg/ml rat-tail collagen type I (Corning, 354236), filtered 1 N NaOH, 10× Dulbecco's modified Eagle medium (DMEM; 11430030, Gibco, Thermo Fisher Scientific) and Biopak water into four-well microculture dishes (500 μl per well). The gels were solidified inside a 37°C and 5% CO_2_ incubator for 20 min before being washed three times for 30 min with explant medium in a solution containing 1× DMEM, GlutaMAX-I CTS (A12860-01, Gibco, Thermo Fisher Scientific), 10% fetal bovine serum (F7524-1654682, Sigma-Aldrich) and a 1× solution of penicillin (10 U/ml), streptomycin (10 mg/ml; P4333, Sigma-Aldrich) and fungizone (250 µg/ml, SV30078.01, Hyclone). The gel was pre-soaked using explant medium containing the drug of interest. Drugs are listed in Table S3. The following day, OFTs were dissected from E11.5 embryos in fresh 1× DMEM, opened, placed with the endocardium side lying face down on gel and allowed to attach for 24 h at 37°C and 5% CO_2_. The next day, we added 500 μl of explant medium, containing the required drug and 1% insulin-transferrin-selenium (ITS; 51500056, Gibco, Thermo Fisher Scientific). After 3 days in culture, explants were fixed in 4% PFA and immunofluorescence was performed. Statistical analyses were performed using unpaired two-tailed Student's *t*-test. Data are presented as mean±s.d.

### Microscopy and confocal imaging

Brightfield imaging was performed using an Olympus BX51 Microscope and Olympus cellSense software. Confocal images were acquired using a Nikon A1-R confocal microscope and Zeiss LSM700 confocal microscope. Confocal data were processed using the ZEN 2012 software (black edition) and image analysis was performed using the Fiji image software. Images were processed in Adobe Photoshop Creative Suite 5.1.

### Echocardiographic recordings

LV function and wall thickness were analyzed by transthoracic echocardiography in 30-week-old male mice. Mice were mildly anesthetized by inhalation of isoflurane/oxygen (1-2%/98.75%), adjusted to obtain a target heart rate of 450±50 beats per minute, and examined with a 30 MHz transthoracic echocardiography probe. Images were obtained with a Vevo 2100 imaging system (VisualSonics). LV long-axis M-mode views were obtained as described previously (Cruz-Adalia et al., 2010). From these images, LV mass, LV systolic volume and LV diastolic volume were measured, LV systolic function was assessed by estimating the LV shortening fraction and LV ejection fraction, and the percentage of strain was assessed by measuring the percentage of ascendant aorta deformation by estimating the diameter in diastole and systole of the aortic arch (Cruz-Adalia et al., 2010). Statistical analyses were performed using unpaired two-tailed Student's *t*-test. Data are presented as mean±s.d.

### RNA-seq

RNA was isolated from E11.5 *Gata6^STOP/+^* and control OFTs. Samples were distributed in three pools of four pairs of OFT per genotype. Tissue was homogenized with a pestle mechanical homogenizer and RNA was extracted with an Arcturus PicoPure RNA Isolation kit (Thermo Fisher Scientific, KIT0214). RNA libraries were prepared using the NEBNext Ultra II Directional RNA Library Prep Kit (New England Biolabs) and sequenced in a Nextseq 2000 Illumina sequencer using a 60 bp single-end elongation protocol. Sequenced reads were checked for quality control and pre-processed using Cutadapt v1.18 (Kechin et al., 2017) to remove adapter contaminants. Resulting reads were aligned and gene expression quantified using RSEM v1.2.3 (Li and Dewey, 2011) over mouse reference GRCm38 with Ensembl genebuild. Differential gene expression was analyzed with the EdgeR R package (v3.32.1 on R 4.0.3) (Robinson et al., 2010). Genes with one count per million in at least three samples were defined as expressed and retained for later analysis. Counts were normalized by the trimmed mean of M-values (TMM) method. Differential gene expression was tested using a generalized linear model as implemented in the EdgeR package. Genes showing altered expression with an adjusted *P*-value <0.05 were considered differentially expressed. The set of differentially expressed genes was used for functional analysis with Ingenuity Pathway Analysis Software (Qiagen-IPA) (Krämer et al., 2014), where we used Benjamini–Hochberg adjusted *P*-value <0.05 for significance. GSEA was performed on the complete set of expressed genes, against the Hallmark term database. A nominal *P*-value <0.1 was used to select for significantly enriched gene sets.

### Statistical analysis

Sample sizes, statistical tests and *P*-values are specified in the corresponding figure legends and corresponding subsections of the Materials and Methods. For comparisons between two groups, mean±s.d. is represented and unpaired two-tailed Student's *t*-test was performed. For experiments comparing two groups of categorical variables, mean±s.d. per group is represented and a Fisher's exact test was performed. Differences were considered statistically significant at *P*<0.05 (Table S4). Statistical analysis and graphical representation were performed using GraphPad Prism v8.

## Supplementary Material

10.1242/DMM.050934_sup1 Supplementary information

Table S1. Lethality Table Sheet 1: *Gata6^flox/flox^Nkx2.5^Cre^*

Table S2. RNA-seq of E11.5 *Gata6^STOP/+^* OFT Sheet 1: raw and normalized gene expression, annotations and differential expression analysis results for all genes.
Sheet 2: differentially expressed (DE) genes (adj p-val<0.05), 113 total, 53 upregulated, 60 down-regulated.
Sheet 3: Panther enrichment results for the collection of 113 DE genes, against the Biological Process GO term database, diseases and functions.
Sheet 4: GSEA Hallmark gene sets with NOM p-val<0.1.

Table S3. Resources of materials and methods Sheet 1: CRISPR-Cas9 reagents.
Sheet 2: Microinjection summary
Sheet 3: Primers Genotyping
Sheet 4: Probe
Sheet 5: Antibodies
Sheet 6: Drug concentration

Table S4. Statistics Organized per Figure and Figures S.
