# Supplementary material for: Deficient GATA6–CXCR7 signaling leads to bicuspid aortic valve
Source: Dis Model Mech. 2024 Sep 10;17(9):dmm050934. doi: 10.1242/dmm.050934 (PMC11413932; doi:10.1242/dmm.050934)
Supplement: Supplementary information [file DMM-17-050934-s1.pdf]

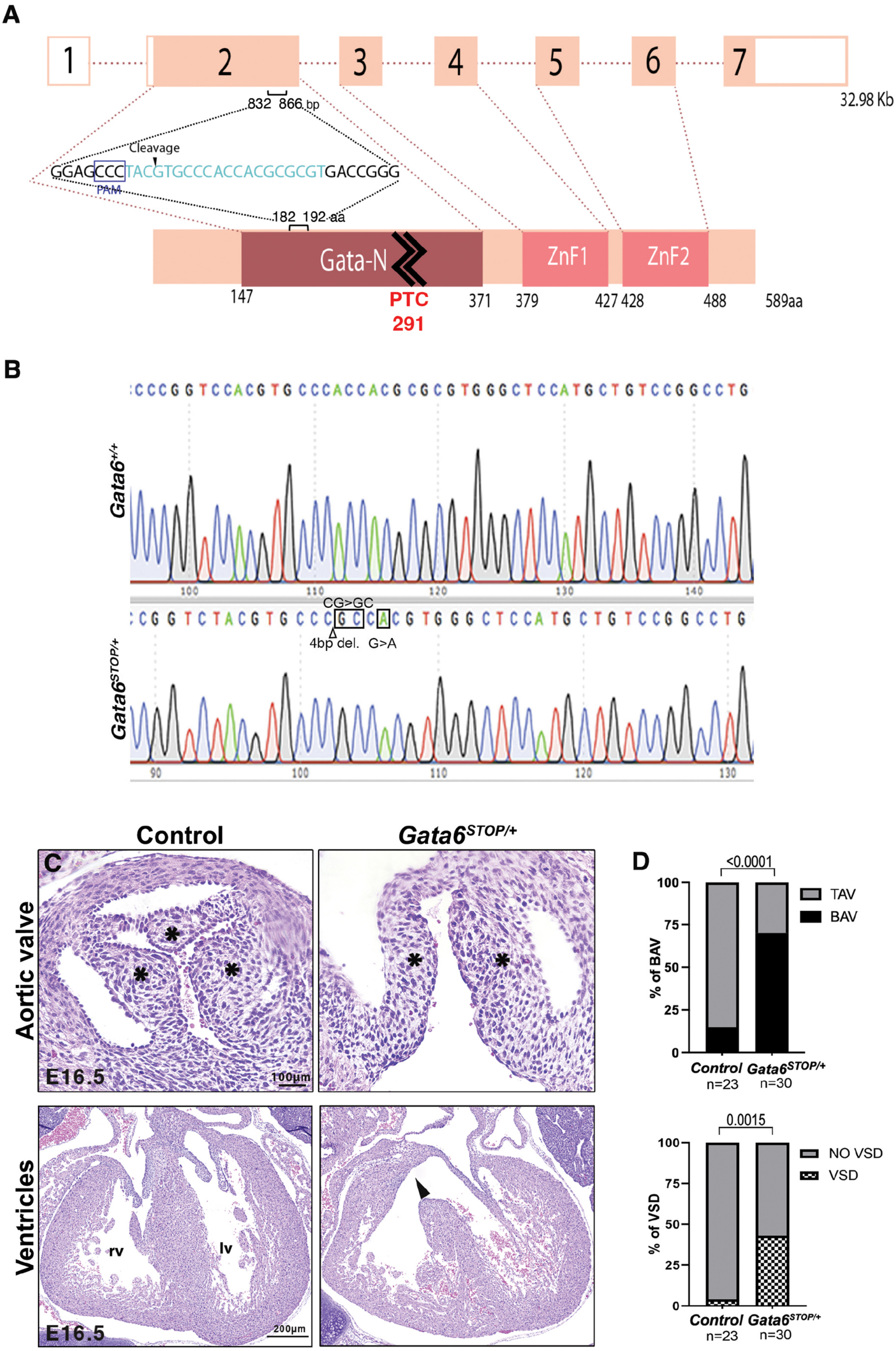

Disease Models & Mechanisms • Supplementary information

Fig. S1. Generation of *Gata6*$^{STOP/+}$ mice by CRISPR-Cas9. (A) Diagram of *Gata6* mouse gene organization and relevant protein domains (Gata-N and ZnF). The sequence of the crRNA containing the PAM site recognized by the Cas9 enzyme is shown. The frameshift mutation results in a premature termination codon (PTC) at aminoacid 291. **(B)** Sequencing of the targeted region of *Gata6*$^{STOP/+}$ and WT pups confirms a 4 bp deletion (from bp 849 to 852) of the cDNA (arrowhead). Also indicated are the 2 bp substitution GC>CG 853,854 (first square) and the 1 bp substitution G>A 856 (second square). Bp, base pairs. **(C)** H&E staining **of** E16.5 *Gata6*$^{STOP/+}$ and control of aortic valve and ventricle sections. **(D)** Quantification of % of BAV and VSD. Asterisks indicate the position of the leaflets. The arrowhead indicates VSD. rv, right ventricle. lv, left ventricle. p-values obtained by Fisher´s Exact test. n (number of embryos)=23 control, n=30 *Gata6*$^{STOP/+}$ embryos.

Disease Models & Mechanisms • Supplementary information

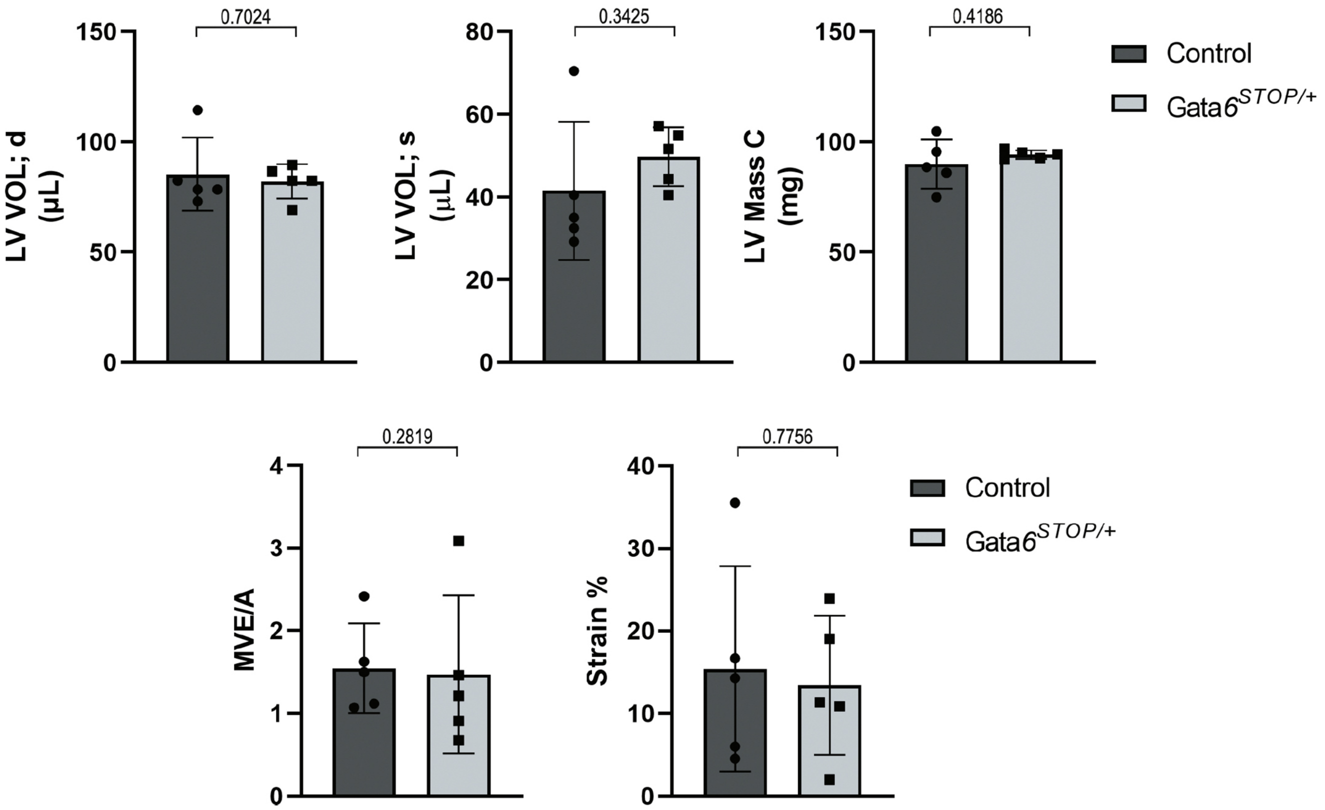

Fig. S2. Cardiac function parameters in 30 weeks-old *Gata6*$^{STOP/+}$ mice. Left ventricle diastolic volume (LV VOL; d), left ventricle systolic volume (LV VOL; s), left ventricle mass (LV Mass C) measurements. The ratio of the early (E) to late (A) ventricular filling velocities of mitral valve (MVE/A) for diastolic dysfunction. Strain %. Data are represented as means ± SD. p-values obtained by Student's t-test. n, number of mice. n=5 control, n=5 *Gata6*$^{STOP/+}$ mice.

Disease Models & Mechanisms • Supplementary information

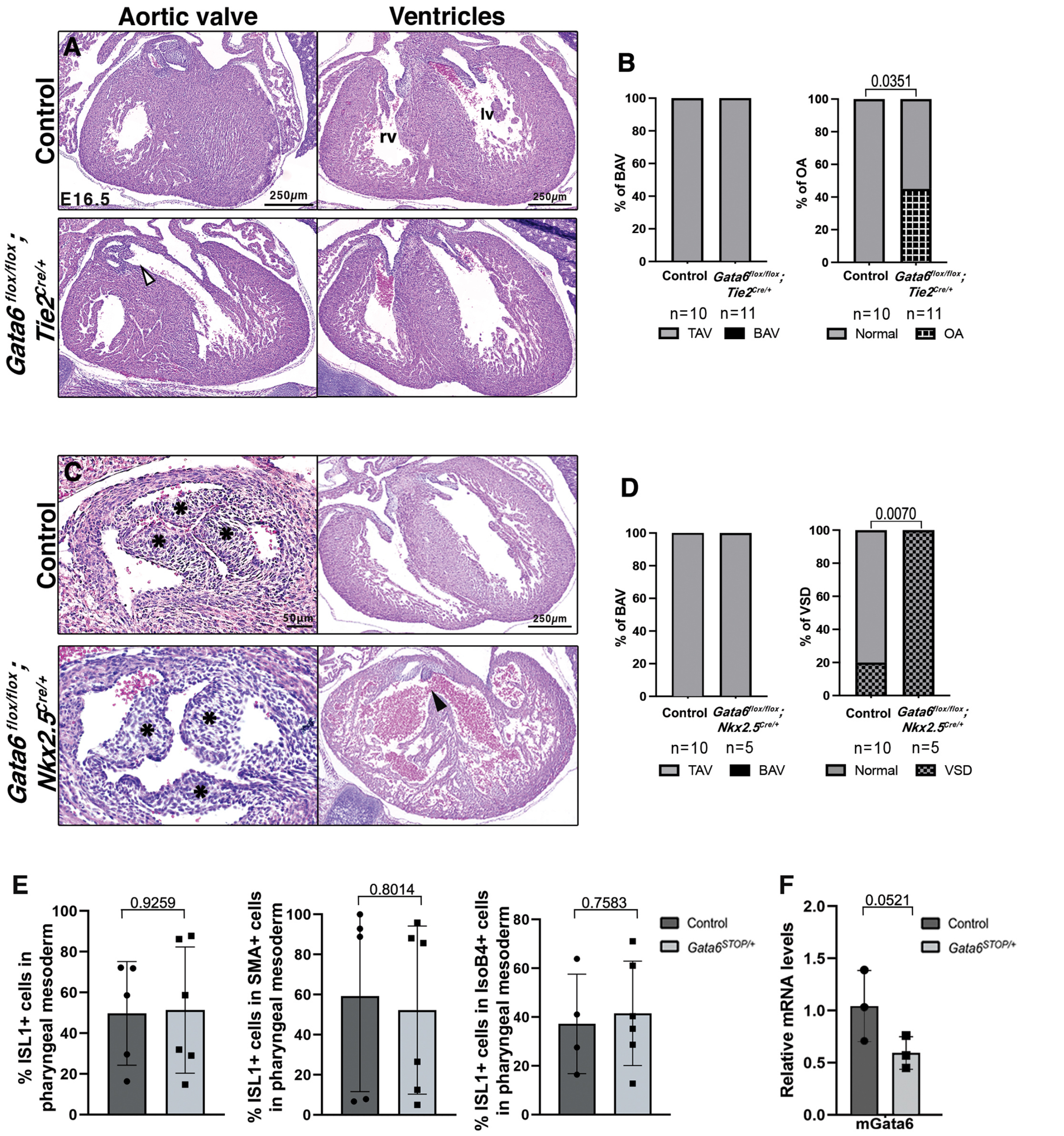

Fig. S3. Deleting GATA6 in endocardium/endothelium or myocardium does not recapitulate the *Gata6*$^{STOP/+}$ phenotype. (A) H&E staining of E16.5 *Gata6*$^{flox/flox}$*; Tie2*^{Cre} and control aortic valve and ventricle sections. The white arrowhead points to the overriding aorta (OA). rv, right ventricle. lv, left ventricle. **(B)** Quantification of % BAV and OA. n=10 control, n=11 *Gata6*$^{flox/flox}$*; Tie2*^{Cre} embryos. **(C)** H&E staining of E16.5 *Gata6*$^{flox/flox}$*;Nkx2.5*^{Cre} and control aortic valve sections and ventricle sections. (**D)** Quantification of % BAV and ventricular septal defect (VSD). Asterisks indicate the position of the leaflets. The arrowhead points to the VSD. p-values obtained by Fisher's Exact test. n=10 control, n=5 *Gata6*$^{flox/flox}$*;Nkx2.5*^{Cre} embryos. **(E)** Quantification of % ISL1+ cells in total, SMA+ and IsoB4+ cells in the pharyngeal mesoderm. Data are represented as means ± SD. p-values obtained by Student's t-test. n=5 control, n=6 *Gata6*$^{STOP/+}$, number of embryos. **(F)** qRT-PCR analysis of Gata6 expression (relative to Gapdh expression as housekeeping gene) in E11.5 *Gata6*$^{STOP/+}$ and control OFT. Data are represented as means ± SD. p-values obtained by Student's t-test. n=3, number of embryos.

Disease Models & Mechanisms • Supplementary information

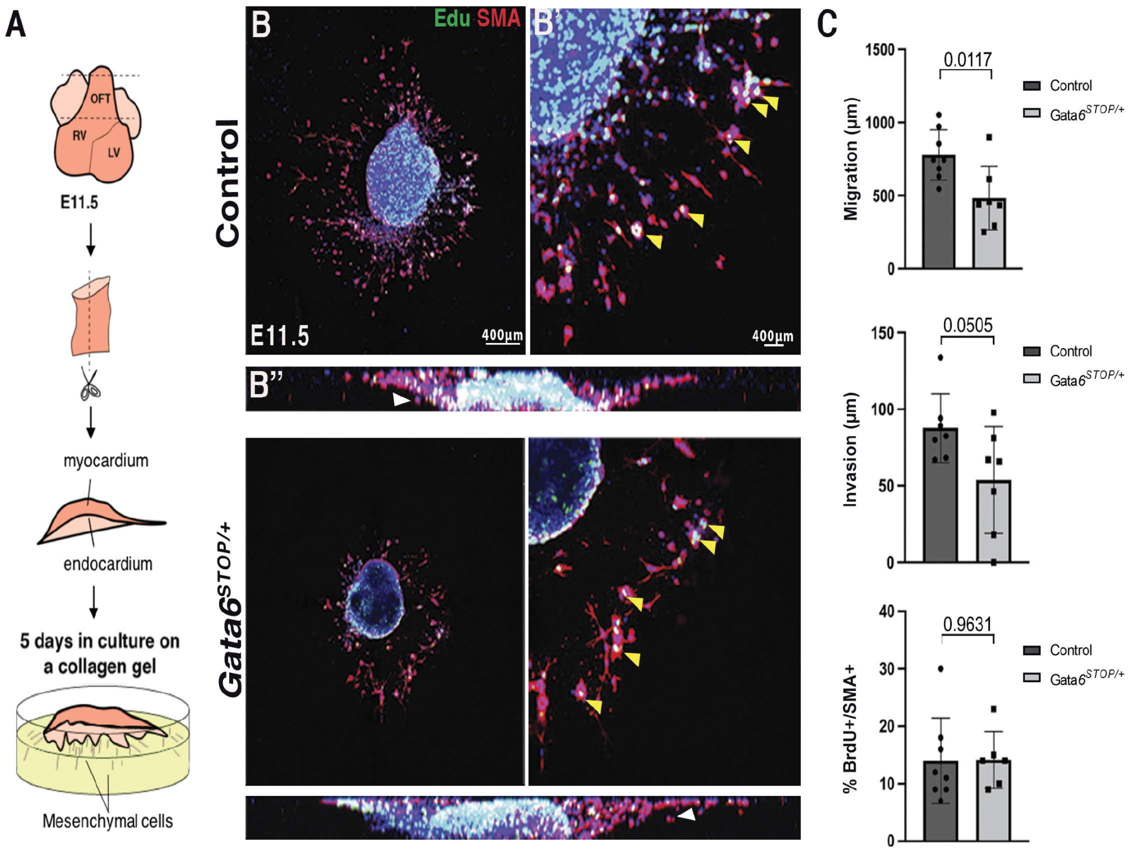

Fig. S4. Impaired migration and invasion in E11.5 *Gata6*$^{STOP/+}$ OFT explants. (A) Depiction of the explant assay. OFTs are dissected, opened longitudinally and the endocardial side is placed face down on the collagen gel, where mesenchymal cells can migrate. **(B)** Fluorescence immunostaining of *Gata6*$^{STOP/+}$ and control explants for EdU. Proliferating cells (green) are indicated by yellow arrows. α-SMA demarcates the mesenchyme (red), DAPI, nuclear counterstain (blue). **(B')** High magnification views of boxed areas with of body of the explant and outwardly migrating mesenchymal cells. **(B'')** 2D orthogonal view of the explants showing mesenchymal cell invasion into the collagen gel. White arrowheads indicate invading cells. **(C)** Quantification of mesenchymal migration, invasion and proliferation. Data are represented as means ± SD. p-values were obtained by Student's t-test. n=8 control, n=7 *Gata6*$^{STOP/+}$ OFT explants.

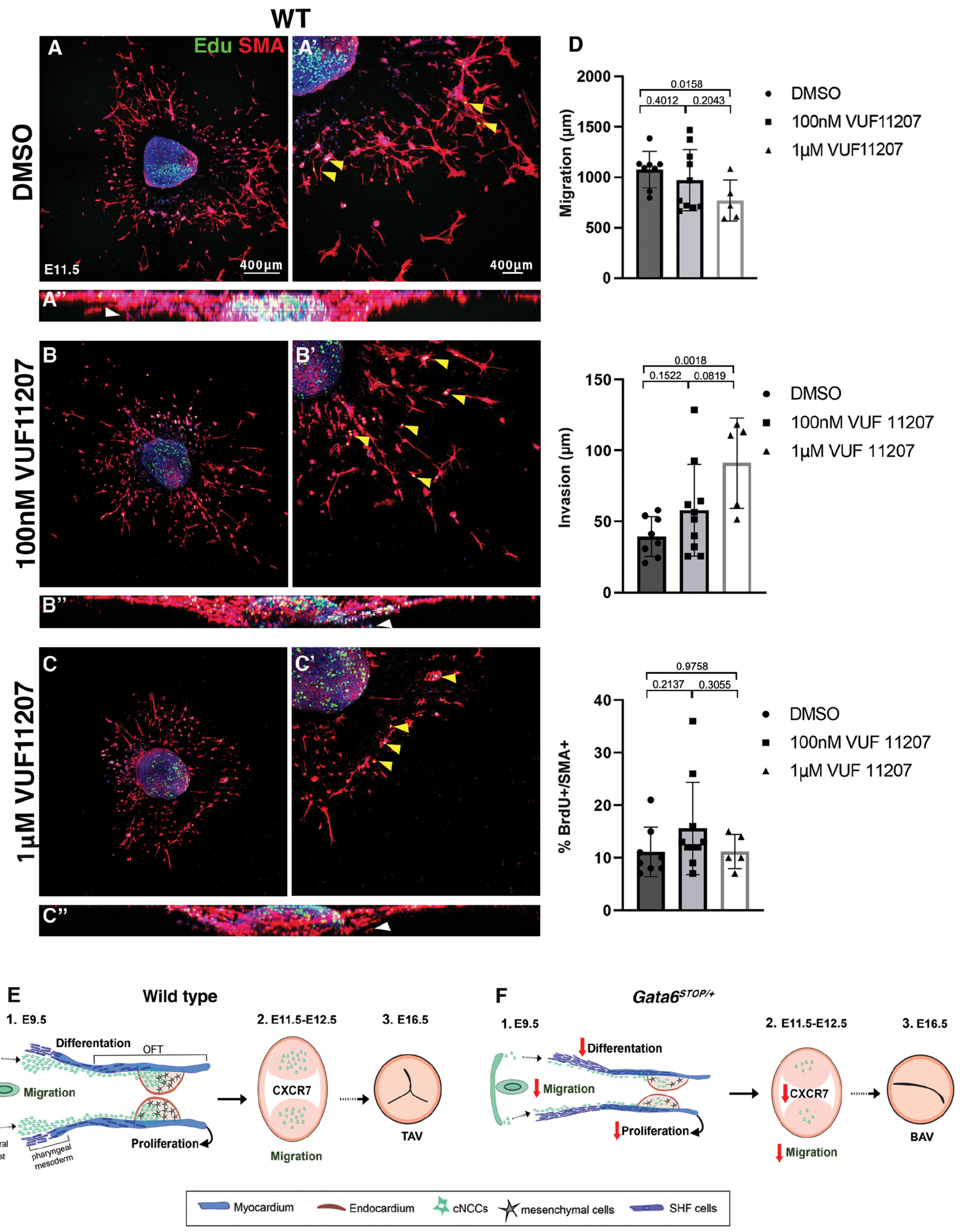
WT
A
Edu SMA
A'
DMSO
E11.5
400µm
400µm
A''
B
B'
100nM VUF11207
B''
C
C'
1µM VUF11207
C''
D
2000
1500
1000
500
0
Migration (µm)
0.0158
0.4012
0.2043
DMSO
100nM VUF11207
1µM VUF11207
150
100
50
0
Invasion (µm)
0.0018
0.1522
0.0819
DMSO
100nM VUF 11207
1µM VUF 11207
40
30
20
10
0
% BrdU+/SMA+
0.9758
0.2137
0.3055
DMSO
100nM VUF 11207
1µM VUF 11207
E
Wild type
1. E9.5
Differentation
OFT
Migration
Neural crest
pharyngeal mesoderm
Proliferation
2. E11.5-E12.5
CXCR7
Migration
3. E16.5
TAV
F
Gata6STOP/+
1. E9.5
Differentation
Migration
Proliferation
2. E11.5-E12.5
CXCR7
Migration
3. E16.5
BAV
Myocardium
Endocardium
cNCCs
mesenchymal cells
SHF cells

Fig. S5. Supplementing OFT explants with a CXCR7 agonist decreases migration and increases invasion. (A-C) Immunofluorescence staining for EdU of E11.5 WT OFT explants treated with carrier solution and indicated amounts of VUF11207. Proliferating cells (green) are indicated by yellow arrows. α-SMA demarcates mesenchyme (red). DAPI, nuclear counterstain (blue). **(A'-C')** High magnification views of boxed areas with of body of the explant and outwardly migrating mesenchymal cells. Yellow arrowheads indicate proliferating cells. **(A''-C'')** 2D orthogonal view of the explants showing mesenchymal cell invasion into the collagen gel. White arrowheads indicate invading cells. **(D)** Quantification of mesenchymal migration, invasion and proliferation. Data are represented as means ± SD. p-values were obtained by Student's t-test. n=8 WT DMSO, n=10 WT 100nM VUF11207, n=5 WT 1 µM VUF11207, OFT explants. (**E,F**) Model for Bicuspid Aortic Valve (BAV) formation in *Gata6*$^{STOP/+}$ mice. (**E**) Wild type. 1. At embryonic day 9.5 (E9.5), second heart field (SHF) endocardial and myocardial progenitor cells originating from the pharyngeal mesoderm, populate the distal outflow tract (OFT). At E10.5-E12.5, cardiac neural crest cells (cNCCs)-derived mesenchyme cells migrate into the distal endocardial cushions, to ensure OFT septation and patterning of aortic and pulmonary valves. The interaction between SHF and cNCCs within the pharyngeal mesoderm is critical for orchestrating OFT development. 2. Downstream of Gata6, Cxcr7 regulates the migration of mesenchymal cells essential for the growth of endocardial cushions. 3. At E16.5, the aortic valve is characterized by three symmetrical leaflets. (**F**) *Gata6*$^{STOP/+}$ mutant. 1. At E9.5, *Gata6*$^{STOP/+}$ mice SHF progenitors' differentiation and proliferation are impaired, resulting in a shorter and narrower OFT. At E12.5, the post-migratory cNCC-derived mesenchyme population is decreased, resulting in defects in OFT septation defects. 2. *Cxcr7* expression in the OFT is below normal, leading to disrupted mesenchymal cell migration and impaired growth of the endocardial cushions. 3. The resulting BAV at E16.5 may arise from impaired lengthening, and mis-patterning of the OFT.

Disease Models & Mechanisms • Supplementary information

Table S1. Lethality Table

Sheet 1: *Gata6$^{flox/flox}$Nkx2.5^{Cre}*

Available for download at
https://journals.biologists.com/dmm/article-lookup/doi/10.1242/dmm.050934#supplementary-data

Table S2. RNA-seq of E11.5 *Gata6$^{STOP/+}$* OFT

Sheet 1: raw and normalized gene expression, annotations and differential expression analysis results for all genes.

Sheet 2: differentially expressed (DE) genes (adj p-val<0.05), 113 total, 53 up-regulated, 60 down-regulated.

Sheet 3: Panther enrichment results for the collection of 113 DE genes, against the Biological Process GO term database, diseases and functions.

Sheet 4: GSEA Hallmark gene sets with NOM p-val<0.1.

Available for download at
https://journals.biologists.com/dmm/article-lookup/doi/10.1242/dmm.050934#supplementary-data

Table S3. Resources of materials and methods Sheet 1: CRISPR-Cas9 reagents.

Sheet 2: Microinjection summary

Sheet 3: Primers Genotyping

Sheet 4: Probe

Sheet 5: Antibodies

Sheet 6: Drug concentration

Available for download at
https://journals.biologists.com/dmm/article-lookup/doi/10.1242/dmm.050934#supplementary-data

Table S4. Statistics

Organized per Figure and Figures S.

Available for download at
https://journals.biologists.com/dmm/article-lookup/doi/10.1242/dmm.050934#supplementary-data

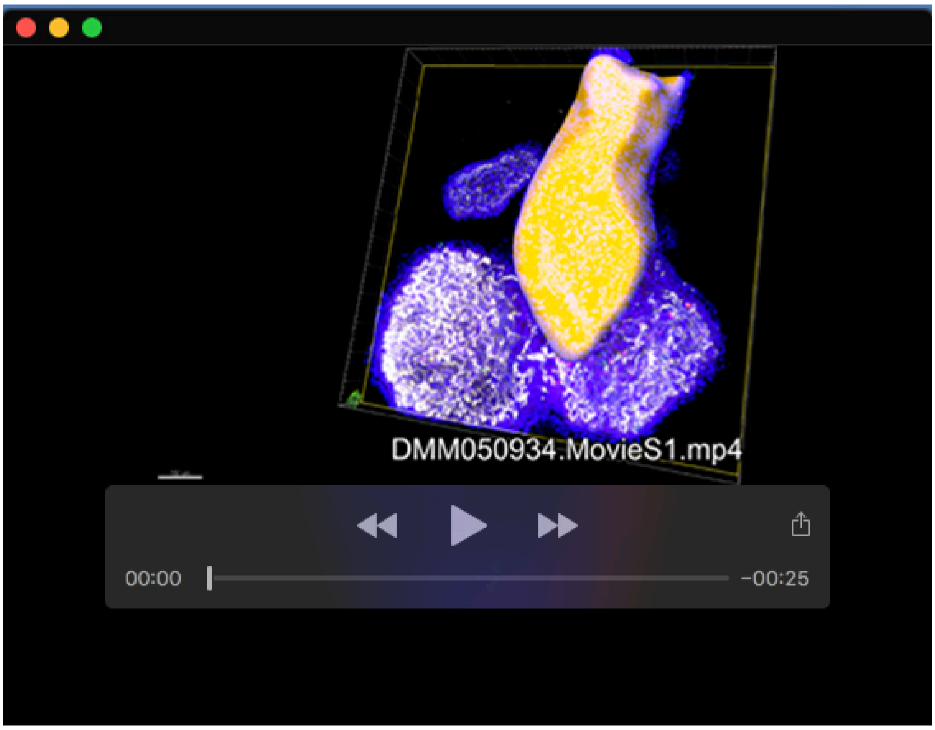

Movie 1. 3D modeling with IMARIS of E11.5 control OFT. Whole mount IF at E11.5 with IsoB4 for endocardium (white) and DAPI for counterstaining (related to Fig. 2A).

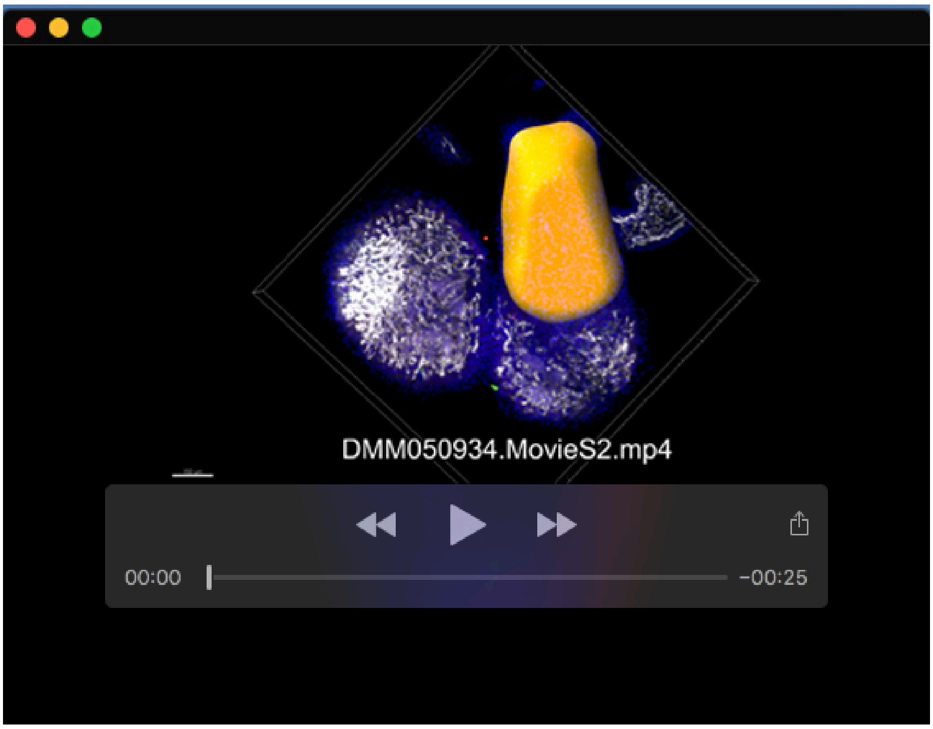

Movie 2. 3D modeling with IMARIS of *Gata6*$^{STOP/+}$ OFT. Whole mount IF at E11.5 with IsoB4 for endocardium (white) and DAPI for counterstaining (related to Fig. 2A).
